# Global Inhibition of DC Priming Capacity in the Spleen of Self-Antigen Vaccinated Mice Requires IL-10

**DOI:** 10.3389/fimmu.2014.00059

**Published:** 2014-02-17

**Authors:** Douglas M. Marvel, Olivera J. Finn

**Affiliations:** ^1^Department of Immunology, University of Pittsburgh School of Medicine, Pittsburgh, PA, USA

**Keywords:** IL-10, dendritic cells, cancer vaccine, MUC1, T cell response

## Abstract

Dendritic cells (DC) in the spleen are highly activated following intravenous vaccination with a foreign-antigen, promoting expansion of effector T cells, but remain phenotypically and functionally immature after vaccination with a self-antigen. Up-regulation or suppression of expression of a cohort of pancreatic enzymes 24–72 h post-vaccination can be used as a biomarker of stimulatory versus tolerogenic DC, respectively. Here we show, using MUC1 transgenic mice and a vaccine based on the MUC1 peptide, which these mice perceive as a self-antigen, that the difference in enzyme expression that predicts whether DC will promote immune response or immune tolerance is seen as early as 4–8 h following vaccination. We also identify early production of IL-10 as a predominant factor that both correlates with this early-time point and controls DC function. Pre-treating mice with an antibody against the IL-10 receptor prior to vaccination results in DC that up-regulate CD40, CD80, and CD86 and promote stronger IFNγ+ T cell responses. This study suggests that transient inhibition of IL-10 prior to vaccination could improve responses to cancer vaccines that utilize self-tumor antigens.

## Introduction

The impact of IL-10 on the cells of the immune system is well studied and varied. Originally identified as cytokine synthesis inhibitory factor, IL-10 can play a role in the development and maturation of almost all immune cells (1, 2). Signaling through the IL-10 receptor (IL-10R) occurs through a STAT3 intermediate and is known to induce SOCS-3 expression, to suppress IFN signaling by blocking STAT1 phosphorylation, and to inhibit NF-κB signaling by preventing its nuclear translocation as well as inhibiting its binding to DNA (2, 3). In dendritic cells (DC), known for being the most important professional antigen presenting cells, IL-10 can reduce expression of MHC Class II and the costimulatory molecules CD80/86 and CD40, as well as reduce IL-12 secretion (3–6). This is true even for DC previously activated with IFNγ. IL-10 can also prevent monocyte differentiation into DC (2).

IL-10 has a profound effect on T cells as well. For example, reduced IL-12 production by DC affected by IL-10 antagonizes the development of T helper type 1 (Th1) responses while reduced MHC II levels on DC result in presentation of low density antigen that preferentially stimulates differentiation of regulatory CD4 T cells (7, 8). IL-10 can also act directly on T cells to inhibit synthesis of cytokines like IL-2 and IFNγ in CD4 T cells or to inhibit their proliferation (3). The effect of IL-10 on CD8 T cells is less clear although some studies have shown that IL-10 can favor activation of CD8 T cells (9–11).

Recently, our group implicated IL-10 in controlling in part the function of DCs post-vaccination with antigens derived from self-proteins. Using the MUC1 transgenic (MUC1.Tg) mouse model and a peptide derived from the extracellular domain of the tumor antigen MUC1, we showed that 24 h following vaccination, there is an IL-10 dependent suppression of DC activation that is detectable via suppression of expression of a newly discovered biomarker: a cohort of pancreatic enzymes. These enzymes, expressed in the spleen only by DC and represented by trypsin 1 and carboxypeptidase B1 (CPB1), are up-regulated post-vaccination with a foreign but not a self-antigen and identified a DC population that has higher MHC Class II, higher costimulatory molecule expression, and a higher T cell stimulatory capacity (12).

In this study, we present new evidence of an important role for IL-10 in the suppression of splenic DC following intravenous vaccination with a self-antigen. We show an early (4–8 h) up-regulation in IL-10 levels in spleens of self-antigen vaccinated mice that is not seen in mice that see that same antigen as foreign and coincides with the time when we also see differences in biomarker enzyme expression. Furthermore, DC in the spleens of self-antigen vaccinated mice have an increased sensitivity to IL-10. When the effect of IL-10 is blocked by pre-vaccination treatment of mice with an anti-IL-10R blocking antibody, there is a significant increase in the activation level and stimulatory capacity of DC at 24 h post-vaccination and a significant increase in CD4 T cell responses 7 days post-vaccination. These data implicate IL-10 in the regulation of antigen-specific immunity versus tolerance at a previously underappreciated early time post-vaccination, and suggest that manipulating its function at the time of vaccination might overcome tolerance and improve responses to cancer vaccines that utilize self-antigens.

## Materials and Methods

### Mice

Human MUC1.Tg mice (13) on the C57Bl/6 background were a generous gift from Dr. Sandra Gendler (Mayo Clinic) and were bred and maintained in the University of Pittsburgh Animal Facility. C57Bl/6 (WT) mice were purchased from The Jackson Laboratory. All experiments were approved by the Institutional Animal Care and Use Committee of the University of Pittsburgh.

### MUC1 vaccination

A 100-aa peptide containing five repeats of the MUC1 VNTR sequence HGVTSAPDTRPAPGSTAPPA, was synthesized as previously described (14) by the University of Pittsburgh Genomics and Proteomics Core Laboratories. For soluble peptide vaccinations, 100 μg of this 100mer peptide admixed with 50 μg polyinosinic–polycytidylic acid and poly-l-lysine (Poly-ICLC; Hiltonol) was brought up to 100 μL with PBS and injected via tail vein. For DC-based vaccinations, DC were prepared as previously described (15). Briefly, RBC lysed bone marrow cells were put into culture for 6 days in AIM-V supplemented with 10 ng/mL GM-CSF (Miltenyi), feeding once on day 3. On day 6, semi-adherent cells were collected by gentle agitation and put into culture overnight in AIM-V containing 33 μg/mL MUC1 100mer peptide and 25 μg/mL Poly-ICLC. The next day, mature DC were collected and resuspended in PBS at a final concentration of 0.5^−1^ × 10^6^ cells/mL. One hundred microliters of this solution was then injected intravenously via tail vein.

### IL-10R blockade

Where indicated, mice were given 250 μg of an antibody against the IL-10R (Bio X Cell, Clone 1B1.3A) or an isotype-matched control antibody (Bio X Cell, Clone HPRN), intraperitoneally. Twenty-four to forty-eight hours following treatment, mice were vaccinated as described in “MUC1 vaccination” above and analyzed as described.

### Quantitative RT-PCR

RNA was extracted from whole spleen using TRIzol (Invitrogen) according to the manufacturer’s protocol. Following extraction, cDNA was generated using oligo(dT) primers and SuperScript III reverse transcriptase (Invitrogen). qPCR was performed using QuantiTect SYBR Green PCR kit (Qiagen) according to the manufacturer’s protocol. Reactions were run on a StepOnePlus instrument (Applied Biosystems). The following primer pairs were used: trypsin 1 (forward: 5′ACTGTGGCTCTGCCCAGCTC3′; reverse: 5′AGCAGGTCTGGTTCAATGACTGT3′), CPB1 (forward: 5′GCCCTGGTGAAAGGTGCAGCAAAGG3′; reverse: 5′AG CCCAGTCGTCAGATCCCCCAGCA3′), IL-10 (forward: 5′CTTC CCAGTCGGCCAGAGCCA3′; reverse: 5′ CTCAGCCGCATCCTG AGGGTCT3′), and HPRT (forward: 5′TGAGCCATTGCTGAGGC GGCGA3′; reverse: 5′CGGCTCGCGGCAAAAAGCGGTC3′).

### Intracellular cytokine staining/flow cytometry

For *ex vivo* T cells assays, 7–9 days post MUC1 vaccination, mice were sacrificed and spleens were removed. Single-cell suspensions were made by mashing the spleens through a 40-μm filter. Total T cells were then bead isolated (Pan T Cell Isolation Kit II, Miltenyi) and cultured with day 6 MUC1-loaded BMDC (prepared as described in “MUC1 vaccination”) for 4–6 h in the presence of GolgiStop (BD biosciences). Cells were then stained with the indicated antibodies using the BD Cytofix/Cytoperm™ kit (BD Bioscience) according to the manufacturer’s protocol. All samples were run on a Fortessa (BD bioscience) flow cytometer and analyzed using FACSDiva (BD Bioscineces) and FlowJo software (Tree Star Inc.). Antibodies used: CD3-PerCP, CD11c-BV421, CD80-FITC, CD86-APC/Cy7, CD40-APC, CD3-PeCy5, CD4-V450, CD8-AF700, IFNγ-PeCy7, TNFα-PE, IL-2-APC, CD44-FITC, CD3-APC/Cy7, and CD8 PerCP.

### Phosphoflow

Twenty-four hours following MUC1 vaccination, splenocytes were harvested as above. Post isolation, cells were put into AIM-V with or without 30 ng/mL IL-10 (PeproTech) for 20 min. At the end of culture, cells were immediately fixed in 1.6% PFA for 10 min at room temperature. After 10 min, four volumes of ice-cold methanol were added and samples were stored at −80°C. At the time of staining cells were put at room temp for 10 min and then immediately spun down and resuspended in flow buffer (PBS containing 1% BSA, 0.02% sodium azide, and 2 nM EDTA). After 10 min incubation at room temperature, cells were spun down and washed with flow buffer twice. Samples were then stained with antibodies against cell surface antigens CD11c, NK1.1, and CD3 and phospho-specific anti-pSAT3 antibody for 1 h at room temperature and prepared for analysis via standard protocol and as described above. Antibodies used: CD11c-Pacific Blue, pSTAT3-AF647, NK1.1-PE, and CD3-APC/Cy7.

### *Ex vivo* DC stimulatory capacity analysis

MUC1 transgenic mice were pretreated with antibodies and vaccinated as in “IL-10R Blockade.” Post-vaccination, DC were bead isolated (CD11c MicroBeads, Miltenyi) from the spleens of the vaccinated animals. These DC were put into culture with bead isolated (CD4 T cell Isolation Kit II, Miltenyi) CFSE stained MUC1 specific VFT CD4 T cells (15) at a ratio of 1 DC to 5 VFT cells in complete DMEM. Twenty-four hours after the start of culture half of the media was removed and saved for cytokine analysis. IL-2 was analyzed by ELISA (BD OptEIA Mouse IL-2 ELISA set, BD) according to the manufacturer’s protocol. The media was replaced with fresh cDMEM and the cultures were allowed to incubate for three more days. T cell proliferation was then analyzed by CFSE dilution.

### ELISPOT

Millipore MultiScreen^®^ Filter Plates (Millipore) were pretreated according to the manufacturer’s instructions using the Mouse IFNg ELISPOT kit (Mabtech). Bead isolated CD4 and CD8 T cells (CD4 T cell Isolation Kit II and CD8α Isolation Kit II, Miltenyi) were cultured as above (see Intracellular Cytokine Staining/Flow Cytometry) with MUC1 pulsed BMDC and analyzed according to the established protocol. DC alone, media alone, and T cells alone were used to establish background cytokine production.

### Statistical analysis

Where appropriate, statistical significance was determined by performing an unpaired Student’s *t*-test. *Denotes a *p*-value <0.05 and **denotes a *p*-value of <0.01. When indicated, to allow for pooling of data from multiple experiments, values have been transformed to account for minor variations in instrument settings and other potential sources of variation (i.e., minor batch to batch variance in DC vaccine prep, etc.). Briefly, all experimental values were divided by the mean value of the control group from the experiment in which they were run. “Relative” values therefore represent a standardized deviance from control.

## Results

### IL-10 expression in the spleen is increased 4–8h post-vaccination with MUC1p as self-antigen and correlates with DC suppression

**Figure 1:**
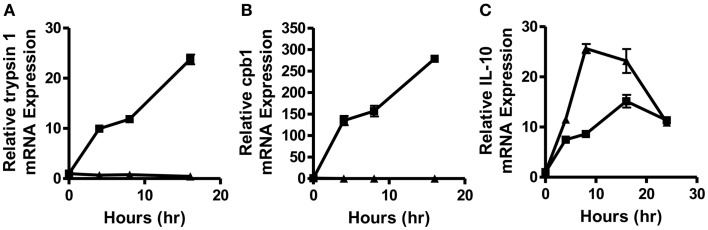
**Splenic DC activation is suppressed as early as 4–8 h post-vaccination with a self-, but not a foreign-antigen and correlated with early IL-10 production in the spleens of these animals**. WT (squares) and MUC1.Tg mice (triangles) were vaccinated with MUC1p plus Poly-ICLC via tail vein. Spleens were removed at indicated hours post-vaccination and total splenic mRNA levels of trypsin 1 **(A)**, carboxypeptidase B1 (CPB1) **(B)**, and IL-10 **(C)** were determined relative to the control gene HPRT. Values shown represent expression relative to the baseline expression in mice of that genotype (WT and MUC1.Tg) at 0 h post-vaccination. Data are representative of three pooled mice were group per time point shown. Data points show mean ± SEM of three technical replicates and are representative of two independent experiments.

In order to determine how quickly post-vaccination with a self- versus a foreign-antigen DC phenotype and function begin to diverge and to obtain a more accurate picture of what factors might be responsible for supporting this divergence, we vaccinated intravenously WT and MUC1.Tg mice with the MUC1 100mer peptide (MUC1p) admixed with the Poly-ICLC adjuvant. MUC1.Tg mice express the human tumor antigen MUC1 under the control of its endogenous promoter and therefore MUC1p is seen as a self-antigen in these mice, whereas it is seen as a foreign-antigen in WT animals. Mice were sacrificed 4, 6, 8, and 16 h post-vaccination and the spleens removed for mRNA isolation and analysis. As early as 4 h post-vaccination, two newly discovered biomarkers of DC activation, trypsin 1 and CPB1 (12), were up-regulated in the spleens of WT mice but suppressed in MUC1.Tg mice (Figures 1A,B). In addition to differences in the levels of these enzymes, which our previous study showed to be expressed only in DC and representative of a larger cohort of “pancreatic” enzymes that robustly activated DC expression, we also detected at this early-time point higher levels of IL-10 mRNA in the spleens of vaccinated MUC1.Tg mice compared to WT mice. At 24 h post-vaccination and later, IL-10 production was at equal levels in self- and foreign-antigen vaccinated mice (Figure 1C and data not shown).

### DC from spleens of MUC1p (self-antigen)-vaccinated MUC1.Tg mice are more sensitive to IL-10 than MUC1p (foreign-antigen)-vaccinated WT mice

**Figure 2:**
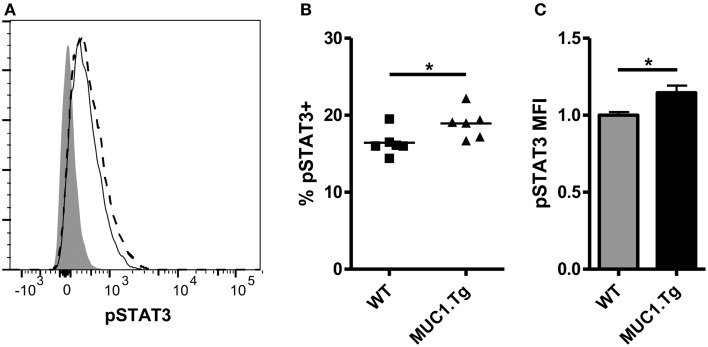
**Dendritic cell from spleens of mice vaccinated with self-antigen have higher levels of phosphorylated STAT3 after IL-10 treatment than DC from spleens of mice vaccinated with foreign-antigen**. WT (solid line) and MUC1.Tg (dashed line) mice were vaccinated with MUC1p via the tail vein. Twenty-four hours post-vaccination splenocytes were removed and treated with 30 ng/mL of IL-10 for 20 min. Following incubation, cells were fixed and phospho-STAT3 expression in CD11c+NK1.1− splenocytes was analyzed via phoshpoflow. **(A)** A representative flow plot is shown. The shaded histogram represents the fluorescence level when cells are treated with standard surface markers and an isotype-matched control instead of the phosphospecific antibody. pSTAT3 positivity **(B)** and MFI **(C)** were analyzed. In **(B)** symbols correspond to individual animals and are representative of two independent experiments. **(C)** Values shown have been normalized to the expression level of the control group (WT) in order to allow for pooling of data from separate experiments run on multiple days. Bars are representative of nine mice from two combined experiments and show the mean ± SEM. *Indicates a *p*-value of <0.05.

The above data showing differences in IL-10 levels early post-vaccination but no difference at 24 h and later would indicate a modest and transient effect by IL-10 on DC. This was, however, inconsistent with our previous observations that functional differences between DC post self-antigen versus foreign-antigen vaccine were evident as late as 72 h post-vaccination (12). We considered the possibility that the early action of IL-10 on DC, along with other factors, might increase their sensitivity to IL-10 at the later-time points. To query this, DC were removed from the spleens of WT and MUC1.Tg mice 24 h post MUC1p vaccination and exposed to IL-10. As signaling through the IL-10R is known to occur through a STAT3 intermediate, the sensitivity of DC to IL-10 was assessed by phosphoflow, measuring phospho-STAT3 levels post *ex vivo* exposure to IL-10. As hypothesized, there was a significant increase in the number of DC showing STAT3 phosphorylation as well as higher levels of pSTAT3 in the spleens of MUC1p-vaccinated MUC1.Tg mice (Figures 2A–C) indicating that DC in the spleens of MUC1.Tg mice are not only exposed to more IL-10 early on, but are also more sensitive to it at the later-time points.

### IL-10R blockade increases costimulatory molecule expression on DC following vaccination with MUC1p as self-antigen

**Figure 3:**
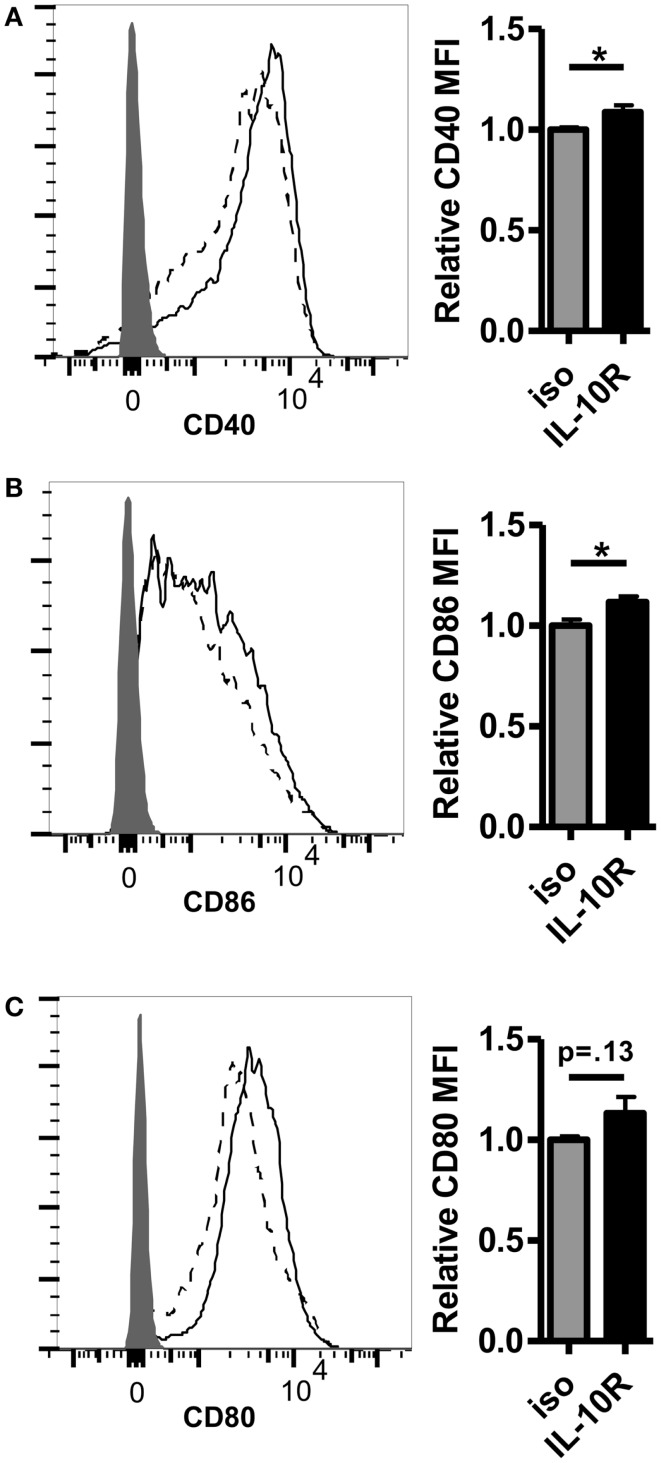
**Pretreatment with an antibody against the IL-10 receptor increases the level of costimulatory molecule expression on DC in the spleens of self-antigen vaccinated mice**. MUC1.Tg mice were pretreated with an antibody against the IL-10 receptor (IL-10R, solid lines) or were given a non-specific isotype control (iso, dashed lines). One to two days later they were vaccinated as in Figure 1 and 24 h post-vaccination, splenocytes were removed and analyzed via flow cytometry. The expression level of CD40 **(A)**, CD86 **(B)**, and CD80 **(C)** on splenic DC (CD11C+, MHC Class II+) was determined. Shaded histograms represent fluorescence in samples stained with isotype alone. Bar graph values shown have been normalized to the expression level of the control group (iso) in order to allow for pooling of data from separate experiments run on multiple days. **(A,C)** Data are combined from two independent experiments and representative of six mice. **(B)** Data are combined from three independent experiments and are representative of 10 mice. Bars represent mean ± SEM. *p*-Values are as stated unless designated by a *, which indicates a *p*-value of <0.05.

**Figure 4:**
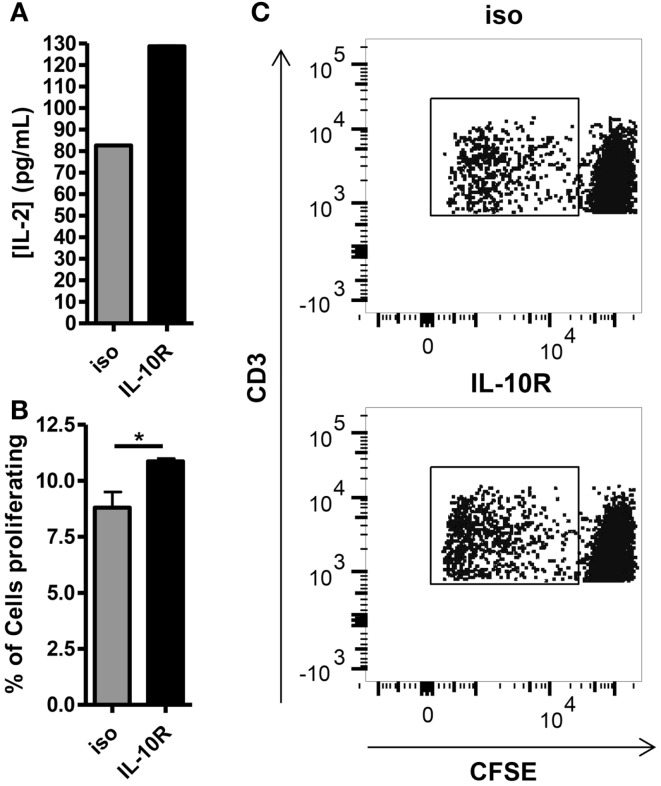
**Blocking of the IL-10 receptor prior to intravenous MUC1 peptide vaccination increases the ability of splenic DC from MUC1.Tg mice to stimulate MUC1 specific CD4 T cells *ex vivo***. MUC1.tg mice were treated as in Figure 3. Twenty-four hours post MUC1 vaccination, splenocytes from three to four mice per treatment group were pooled and bead isolated DC from these pooled splenocytes were put into culture with CFSE labeled MUC1 specific CD4 T cells (VFT cells) at a ratio 1DC:5VFT. Twenty-four hours after the start of culture, half of the culture media was removed and the concentration of IL-2 was measured by ELISA **(A)**. Cultures were allowed to incubate three more days for a total of four and VFT proliferation was analyzed by CFSE dilution **(B,C)**. **(B)** Bars represent the mean percentage of CD3+CD4+ T cells that had proliferated at 4 days of three technical replicates ±SEM. **(C)** A representative flow plot is shown. Data are representative of two to three independent experiments. *Indicates a *p*-value of <0.05.

Given the inverse correlation between IL-10 production and DC pancreatic enzyme expression in the first 24 h following vaccination and previously published data showing that IL-10 is necessary for suppression of trypsin 1 and CPB1 following vaccination with a self-antigen (12), we hypothesized that blocking IL-10 signaling in self-antigen vaccinated mice would improve DC activation and costimulatory molecule expression. We injected MUC1.Tg mice with an antibody against IL-10R and vaccinated intravenously 24–48 h later with MUC1p plus Poly-ICLC. At 24 h post-vaccination, the surface phenotype of splenic DC was analyzed by flow cytometry. As hypothesized, there was an increase in the level of cell surface expression of CD40, CD80, and CD86 in DC from mice pretreated with the antibody to IL-10R, but not from mice treated with the isotype control antibody (Figures 3A–C). Increases in CD40 and CD86 were statistically significant, which is of interest because these two molecules were shown previously to be specifically inhibited in mice vaccinated with a self- but not a foreign-antigen (12). In addition to being less active as measured by surface marker expression, these DC are also less capable of stimulating MUC1 specific CD4 T cells *in vitro*. DC isolated from MUC1.Tg mice pretreated with an antibody against the IL-10R prior to MUC1 vaccination and put into culture with MUC1 specific CD4 T cells induce higher levels of IL-2 (Figure 4A) and CD4 T cell proliferation (Figures 4B,C) compared to DC from MUC1.Tg mice pretreated with an isotype-matched control antibody.

### Blocking IL-10 signaling prior to vaccination with MUC1p as self-antigen improves CD4 T cell response

**Figure 5:**
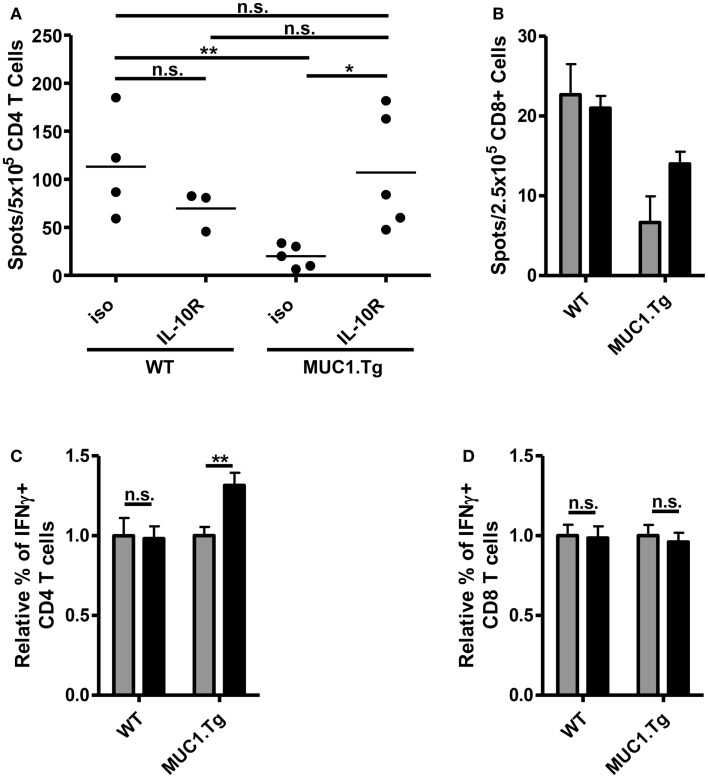
**Treatment with anti-IL-10R antibody at the time of vaccination increases the number of MUC1p specific, IFNγ+ CD4 T cells without an effect on CD8 T cells**. WT and MUC1.Tg mice were pretreated with an antibody against the IL-10 receptor (IL-10R, black bars) or a non-specific isotype control (iso, gray bars). One to two days following antibody treatment, mice were vaccinated with DC loaded with MUC1p. Seven to nine days post-vaccination, spleens were removed and bead isolated CD4 **(A,C)** and CD8 T cells **(B,D)** were cultured with MUC1p loaded bone marrow derived DC overnight and analyzed by ELISPOT **(A,B)**, or were cultured for 6–8 h in the presence of brefeldin-A and analyzed by intracellular flow cytometry **(C,D)**. **(A)** Data are combined from two independent experiments with each spot indicating an individual animal. Data are representative of three independent experiments. **(B)** Bars indicate the average of three technical replicates pooled from three individual animals per group. Data are representative of two independent experiments. **(C,D)** Values shown are normalized to the response of mice of that genotype (WT versus MUC1.Tg) given the control treatment (iso). Data are combined from two independent experiments and are representative of five to six mice per group. Bars represent mean ± SEM. *Indicates a *p*-value of <0.05; **indicates a *p*-value of <0.005.

The increase of costimulatory molecule expression when IL-10 signaling was blocked just prior to vaccination suggested that there would be a resultant increase in the T cell response. To test this, we again pretreated mice with an anti-IL-10R antibody or an isotype-matched control and injected with a vaccine composed of DC loaded with MUC1p. We chose the DC-based vaccine expecting that it would optimally stimulate both CD4 and CD8 T cells, as has been previously shown (16). Seven to nine days post-vaccination, splenic T cells were isolated and their production of relevant cytokines analyzed by ELISPOT and intracellular flow cytometry. In MUC1.Tg mice treated with anti-IL-10R, there was a significant increase in MUC1p specific, IFNγ+ CD4 T cells when compared to mice treated with an isotype-matched control antibody (Figures 5A,C). The level of the response was equivalent to the response of WT mice pretreated with the isotype control antibody (Figure 5A). There was no increase over the isotype control of the T cell response in WT mice pretreated with the anti-IL-10R antibody (Figures 5A,C), indicating that the effect of IL-10 we saw in MUC1.Tg mice was specific for controlling responses to self- but not foreign-antigens. There was a small but not significant increase in the CD8 response that was detectable only by the more sensitive ELISPOT (Figures 5B,D).

## Discussion

Vaccines against cancer have garnered a lot of attention in recent years. Much of this was sparked by the relatively recent approval of Sipuleucel-T, the first vaccine to show survival benefit in a solid metastatic tumor (17, 18). Implementation of Gardasil^®^, a quadrivalent human papilloma virus specific vaccination intended to prevent cervical cancer in women (19, 20) has also sparked new efforts in designing prophylactic cancer vaccines not just for viral cancers but for many tumor types (21–24). Most non-viral tumor antigens fall into the category of self- or altered self-antigens. Mounting an effective immune response against them represents a unique challenge. One must design vaccines that overcome the natural tolerizing forces acting on responses to self-antigens, while minimizing adverse autoimmune effects.

Our work with the MUC1 tumor antigen in the MUC1.Tg mouse model system has shown that hyporesponsiveness to the MUC1 peptide vaccines in these mice is neither due to the elimination of MUC1 peptide-specific T cells by central tolerance, nor solely to their conditioning in the periphery, but rather by the control of their activation (15). Indeed, even when unconditioned MUC1 specific T cells are transferred into MUC1.Tg hosts, they are hyporesponsive to MUC1 peptide vaccination but respond vigorously in WT hosts. Most recently, we determined that the major reason for the lack of T cell response is profound, albeit transient, tolerization of DC in MUC1p-vaccinated MUC1.Tg mice early post-vaccination (12). Here, we show that this is likely due to the very early and exaggerated effect of IL-10 on these DC in the first 4–24 h post-vaccination. IL-10 is known to reduce MHC Class II and costimulatory molecule expression on DC (4–6), DC motility (25, 26), and overall T cell stimulatory capacity (27, 28), all of which are characteristics of DC in the spleens of MUC1p-vaccinated MUC1.Tg mice (12).

The effects of IL-10 on vaccines have been observed previously. In the therapeutic setting, IL-10R blockade alone or along with vaccination can improve Th1 responses and enhance pathogen clearance (29–31). In a prophylactic setting, mice given the BCG vaccination for prevention of *Mycobacterium tuberculosis* show improved Th1 responses and enhanced resistance to pathogen challenge when IL-10R is blocked at the time of vaccination (32). In this paper, we describe a distinct new role for IL-10 in impacting vaccine outcome that is unique in its specificity for self-antigen. The same MUC1 peptide, given as a self-antigen to MUC1.Tg mice but as a foreign-antigen to WT mice, causes only low levels IL-10 production in WT mice and increased production in MUC1.Tg mice. In previously published studies showing improvement in immune responses after IL-10R blockade, IL-10 was produced in response to acute or persistent pathogen infections, whereas in this case it was specifically triggered in response to the presence of a self-antigen or specifically inhibited in the presence of a foreign-antigen.

We have yet to identify the source in the spleen of this early IL-10 production in self-antigen vaccinated mice. Every cell of the immune system can produce IL-10 given proper stimulation. However the kinetics and pattern of IL-10 production in MUC1p-vaccinated MUC1.Tg mice limits the possibilities considerably. The fact that IL-10 production was antigen dependent suggests a cell of the adaptive immune system. Regulatory T cells have previously been shown to be important in preventing MUC1p specific immune responses in MUC1.Tg mice (12, 33). However, preliminary experiments have been unable to identify IL-10 producing regulatory T cells in MUC1.Tg mice at rest or immediately following vaccination (data not shown), as has been shown in some models after self-peptide administration (34). Given that regulatory T cells can modulate the function of a wide variety of innate cells, including NK cells (35, 36) and DCs (37, 38), it is possible that through secretion of another cytokine or through direct interactions, they induce IL-10 production either directly or indirectly in another cell population.

Irrespective of the source, the self-antigen specific role of IL-10 reported in this paper supports IL-10 inhibition as a way of improving the efficacy of vaccines against self-antigens that are candidate tumor antigens. While our major success in this study was in improving CD4 T cell responses, we would hypothesize that CD8 T cell responses generated upon boosting would be improved as well in these animals as a consequence of generation of a larger population of helper CD4 T cells that are required for effective CD8 T cell memory differentiation (39, 40). The concern remains that any manipulation leading to enhanced responses to self/tumor antigens might cause adverse autoimmune reactions. However, current research has shown this concern can be addressed by proper antigen selection. For example, vaccines against self/tumor antigens MUC1 and α-lactalbumin have shown clinical and preclinical efficacy with no induction of autoimmunity (23, 41, 42). And vitiligo, caused by successful anti-melanoma vaccines is an autoimmune event that can be easily tolerated (43–45). Furthermore, while long term IL-10 deficiency can cause adverse autoimmune effects (46, 47), our data suggest that in order to improve the vaccine response, IL-10 would need to be blocked only transiently at the time of initial vaccination.

## Author Contributions

The research reported in this article was conducted by Douglas M. Marvel. Douglas M. Marvel and Olivera J. Finn jointly designed the experiments and prepared the manuscript.

## Conflict of Interest Statement

The authors declare that the research was conducted in the absence of any commercial or financial relationships that could be construed as a potential conflict of interest.

## References

[1] Moore KW O’garra A Malefyt RW Vieira P Mosmann TR Interleukin-10. Annu Rev Immunol (1993) 11:165–90 10.1146/annurev.iy.11.040193.001121 8386517

[2] Moore KW De Waal Malefyt R Coffman RL O’garra A Interleukin-10 and the interleukin-10 receptor. Annu Rev Immunol (2001) 19:683–765 10.1146/annurev.immunol.19.1.683 11244051

[3] Asadullah K Sterry W Volk HD Interleukin-10 therapy – review of a new approach. Pharmacol Rev (2003) 55:241–69 10.1124/pr.55.2.4 12773629

[4] De Smedt T Van Mechelen M De Becker G Urbain J Leo O Moser M Effect of interleukin-10 on dendritic cell maturation and function. Eur J Immunol (1997) 27:1229–35 10.1002/eji.1830270526 9174615

[5] Steinbrink K Wolfl M Jonuleit H Knop J Enk AH Induction of tolerance by IL-10-treated dendritic cells. J Immunol (1997) 159:4772–80 9366401

[6] Brossart P Zobywalski A Grünebach F Behnke L Stuhler G Reichardt VL Tumor necrosis factor α and CD40 ligand antagonize the inhibitory effects of interleukin 10 on T-cell stimulatory capacity of dendritic cells. Cancer Res (2000) 60:4485–92 10969796

[7] Turner MS Kane LP Morel PA Dominant role of antigen dose in CD4+Foxp3+ regulatory T cell induction and expansion. J Immunol (2009) 183:4895–903 10.4049/jimmunol.0901459 19801514 PMC3142864

[8] Gottschalk RA Corse E Allison JP TCR ligand density and affinity determine peripheral induction of Foxp3 in vivo. J Exp Med (2010) 207:1701–11 10.1084/jem.20091999 20660617 PMC2916126

[9] Mocellin S Marincola FM Young HA Interleukin-10 and the immune response against cancer: a counterpoint. J Leukoc Biol (2005) 78:1043–51 10.1189/jlb.0705358 16204623

[10] Brooks DG Walsh KB Elsaesser H Oldstone MBA IL-10 directly suppresses CD4 but not CD8 T cell effector and memory responses following acute viral infection. Proc Natl Acad Sci U S A (2010) 107:3018–23 10.1073/pnas.0914500107 20133700 PMC2840337

[11] Emmerich J Mumm JB Chan IH Laface D Truong H Mcclanahan T IL-10 directly activates and expands tumor-resident CD8+ T cells without de novo infiltration from secondary lymphoid organs. Cancer Res (2012) 72:3570–81 10.1158/0008-5472.CAN-12-0721 22581824

[12] Farkas AM Marvel DM Finn OJ Antigen choice determines vaccine-induced generation of immunogenic versus tolerogenic dendritic cells that are marked by differential expression of pancreatic enzymes. J Immunol (2013) 190:3319–27 10.4049/jimmunol.1203321 23420890 PMC3608825

[13] Rowse GJ Tempero RM Vanlith ML Hollingsworth MA Gendler SJ Tolerance and immunity to MUC1 in a human MUC1 transgenic murine model. Cancer Res (1998) 58:315–21 9443411

[14] Turner MS Cohen PA Finn OJ Lack of effective MUC1 tumor antigen-specific immunity in MUC1-transgenic mice results from a Th/T regulatory cell imbalance that can be corrected by adoptive transfer of wild-type Th cells. J Immunol (2007) 178:2787–93 17312122 10.4049/jimmunol.178.5.2787

[15] Ryan SO Turner MS Gariepy J Finn OJ Tumor antigen epitopes interpreted by the immune system as self or abnormal-self differentially affect cancer vaccine responses. Cancer Res (2010) 70:5788–96 10.1158/0008-5472.CAN-09-4519 20587526 PMC2905500

[16] Soares MM Mehta V Finn OJ Three different vaccines based on the 140-amino acid MUC1 peptide with seven tandemly repeated tumor-specific epitopes elicit distinct immune effector mechanisms in wild-type versus MUC1-transgenic mice with different potential for tumor rejection. J Immunol (2001) 166:6555–63 11359807 10.4049/jimmunol.166.11.6555

[17] Kantoff PW Higano CS Shore ND Berger ER Small EJ Penson DF Sipuleucel-T immunotherapy for castration-resistant prostate cancer. N Engl J Med (2010) 363:411–22 10.1056/NEJMoa1001294 20818862

[18] Boikos SA Antonarakis ES Immunotherapy for prostate cancer enters its golden age. Clin Med Insights Oncol (2012) 6:263–73 10.4137/CMO.S7475 22844202 PMC3403579

[19] McCormack PL Joura EA Quadrivalent human papillomavirus (types 6, 11, 16, 18) recombinant vaccine (Gardasil(R)): a review of its use in the prevention of premalignant genital lesions, genital cancer and genital warts in women. Drugs (2010) 70:2449–74 10.2165/11204920-000000000-00000 21142263

[20] McCormack PL Joura EA Spotlight on quadrivalent human papillomavirus (types 6, 11, 16, 18) recombinant vaccine (Gardasil(R)) in the prevention of premalignant genital lesions, genital cancer, and genital warts in women. BioDrugs (2011) 25:339–43 10.2165/11205060-000000000-00000 21942919

[21] Czerniecki BJ Roses RE Koski GK Development of vaccines for high-risk ductal carcinoma in situ of the breast. Cancer Res (2007) 67:6531–4 10.1158/0008-5472.CAN-07-0878 17638860

[22] Kimura T Finn OJ MUC1 immunotherapy is here to stay. Expert Opin Biol Ther (2013) 13:35–49 10.1517/14712598.2012.725719 22998452

[23] Kimura T Mckolanis JR Dzubinski LA Islam K Potter DM Salazar AM MUC1 vaccine for individuals with advanced adenoma of the colon: a cancer immunoprevention feasibility study. Cancer Prev Res (2013) 6:18–26 10.1158/1940-6207.CAPR-12-0275 23248097 PMC3536916

[24] Liao JB Disis ML Therapeutic vaccines for ovarian cancer. Gynecol Oncol (2013) 130:667–73 10.1016/j.ygyno.2013.06.023 23800697

[25] Wang B Amerio P Sauder DN Role of cytokines in epidermal Langerhans cell migration. J Leukoc Biol (1999) 66:33–9 10410987 10.1002/jlb.66.1.33

[26] Demangel C Bertolino P Britton WJ Autocrine IL-10 impairs dendritic cell (DC)-derived immune responses to mycobacterial infection by suppressing DC trafficking to draining lymph nodes and local IL-12 production. Eur J Immunol (2002) 32:994–1002 10.1002/1521-4141(200204)32:43.3.CO;2-Y 11920565

[27] Macatonia SE Doherty TM Knight SC O’garra A Differential effect of IL-10 on dendritic cell-induced T cell proliferation and IFN-gamma production. J Immunol (1993) 150:3755–65 8097224

[28] Commeren DL Van Soest PL Karimi K Löwenberg B Cornelissen JJ Braakman E Paradoxical effects of interleukin-10 on the maturation of murine myeloid dendritic cells. Immunology (2003) 110:188–96 10.1046/j.1365-2567.2003.01730.x 14511232 PMC1783045

[29] Murray HW Lu CM Mauze S Freeman S Moreira AL Kaplan G Interleukin-10 (IL-10) in experimental visceral leishmaniasis and IL-10 receptor blockade as immunotherapy. Infect Immun (2002) 70:6284–93 10.1128/IAI.70.11.6284-6293.2002 12379707 PMC130311

[30] Murray HW Moreira AL Lu CM DeVecchio JL Matsuhashi M Ma X Determinants of response to interleukin-10 receptor blockade immunotherapy in experimental visceral leishmaniasis. J Infect Dis (2003) 188:458–64 10.1086/376510 12870129

[31] Brooks DG Lee AM Elsaesser H Mcgavern DB Oldstone MBA IL-10 blockade facilitates DNA vaccine-induced T cell responses and enhances clearance of persistent virus infection. J Exp Med (2008) 205:533–41 10.1084/jem.20071948 18332180 PMC2275377

[32] Pitt JM Stavropoulos E Redford PS Beebe AM Bancroft GJ Young DB Blockade of IL-10 signaling during Bacillus Calmette-Guérin vaccination enhances and sustains Th1, Th17, and innate lymphoid IFN-γ and IL-17 responses and increases protection to mycobacterium tuberculosis infection. J Immunol (2012) 189:4079–87 10.4049/jimmunol.1201061 22972927 PMC3467194

[33] Sugiura D Denda-Nagai K Takashima M Murakami R Nagai S Takeda K Local effects of regulatory T cells in MUC1 transgenic mice potentiate growth of MUC1 expressing tumor cells in vivo. PLoS One (2012) 7:e44770 10.1371/journal.pone.0044770 23028615 PMC3444443

[34] Burkhart C Liu GY Anderton SM Metzler B Wraith DC Peptide-induced T cell regulation of experimental autoimmune encephalomyelitis: a role for IL-10. Int Immunol (1999) 11:1625–34 10.1093/intimm/11.10.1625 10508180

[35] Kerdiles Y Ugolini S Vivier E T cell regulation of natural killer cells. J Exp Med (2013) 210:1065–8 10.1084/jem.20130960 23733834 PMC3674696

[36] Pedroza-Pacheco I Madrigal A Saudemont A Interaction between natural killer cells and regulatory T cells: perspectives for immunotherapy. Cell Mol Immunol (2013) 10:222–9 10.1038/cmi.2013.2 23524654 PMC4012769

[37] Houot R Perrot I Garcia E Durand I Lebecque S Human CD4+CD25 high regulatory T cells modulate myeloid but not plasmacytoid dendritic cells activation. J Immunol (2006) 176:5293–8 16621995 10.4049/jimmunol.176.9.5293

[38] Veldhoen M Moncrieffe H Hocking RJ Atkins CJ Stockinger B Modulation of dendritic cell function by naive and regulatory CD4+ T cells. J Immunol (2006) 176:6202–10 16670330 10.4049/jimmunol.176.10.6202

[39] Sun JC Bevan MJ Defective CD8 T cell memory following acute infection without CD4 T cell help. Science (2003) 300:339–42 10.1126/science.1083317 12690202 PMC2778341

[40] Williams MA Bevan MJ Effector and memory CTL differentiation. Annu Rev Immunol (2007) 25:171–92 10.1146/annurev.immunol.25.022106.141548 17129182

[41] Finn OJ Forni G Prophylactic cancer vaccines. Curr Opin Immunol (2002) 14:172–7 10.1016/S0952-7915(02)00317-5 11869888

[42] Flemming A Cancer: steps towards a prophylactic breast cancer vaccine. Nat Rev Drug Discov (2010) 9:594–5 10.1038/nrd3233 20671760

[43] Overwijk WW Lee DS Surman DR Irvine KR Touloukian CE Chan C-C Vaccination with a recombinant vaccinia virus encoding a “self” antigen induces autoimmune vitiligo and tumor cell destruction in mice: requirement for CD4+ T lymphocytes. Proc Natl Acad Sci U S A (1999) 96:2982–7 10.1073/pnas.96.6.2982 10077623 PMC15881

[44] van Elsas A Hurwitz AA Allison JP Combination immunotherapy of B16 melanoma using anti-cytotoxic T lymphocyte-associated antigen 4 (Ctla-4) and granulocyte/macrophage colony-stimulating factor (Gm-Csf)-producing vaccines induces rejection of subcutaneous and metastatic tumors accompanied by autoimmune depigmentation. J Exp Med (1999) 190:355–66 10430624 10.1084/jem.190.3.355 PMC2195583

[45] Byrne KT Côté AL Zhang P Steinberg SM Guo Y Allie R Autoimmune melanocyte destruction is required for robust CD8+ memory T cell responses to mouse melanoma. J Clin Invest (2011) 121:1797–809 10.1172/JCI44849 21540555 PMC3083789

[46] Kotlarz D Beier R Murugan D Diestelhorst J Jensen O Boztug K Loss of interleukin-10 signaling and infantile inflammatory bowel disease: implications for diagnosis and therapy. Gastroenterology (2012) 143:347–55 10.1053/j.gastro.2012.04.045 22549091

[47] Shah N Kammermeier J Elawad M Glocker E-O Interleukin-10 and interleukin-10-receptor defects in inflammatory bowel disease. Curr Allergy Asthma Rep (2012) 12:373–9 10.1007/s11882-012-0286-z 22890722

